# Paradoxical roles of caspase-3 in regulating cell survival, proliferation, and tumorigenesis

**DOI:** 10.1083/jcb.202201159

**Published:** 2022-05-12

**Authors:** Ebrahim Eskandari, Connie J. Eaves

**Affiliations:** 1 Terry Fox Laboratory, British Columbia Cancer Agency, Vancouver, British Columbia, Canada; 2 Department of Medical Genetics, University of British Columbia, Vancouver, British Columbia, Canada; 3 School of Biomedical Engineering, University of British Columbia, Vancouver, British Columbia, Canada

## Abstract

Caspase-3 is a widely expressed member of a conserved family of proteins, generally recognized for their activated proteolytic roles in the execution of apoptosis in cells responding to specific extrinsic or intrinsic inducers of this mode of cell death. However, accumulating evidence indicates that caspase-3 also plays key roles in regulating the growth and homeostatic maintenance of both normal and malignant cells and tissues in multicellular organisms. Given that yeast possess an ancestral caspase-like gene suggests that the caspase-3 protein may have acquired different functions later during evolution to better meet the needs of more complex multicellular organisms, but without necessarily losing all of the functions of its ancestral yeast precursor. This review provides an update on what has been learned about these interesting dichotomous roles of caspase-3, their evolution, and their potential relevance to malignant as well as normal cell biology.

## Teaser

Eskandari and Eaves review evidence of caspase-3-regulated protein quality, proliferation, differentiation, and tumorigenic activity in viable cells.

## Structure and regulation of caspase-3

Caspase-3 is a member of a family of Cysteine-ASPartic proteASES (cysteine proteases) best known for their ability to mediate the cleavage of specific target proteins. Caspases are all produced initially as inactive zymogens (called procaspases) that are then subject to activation by a wide range of specific internal and/or external signals. The molecular structure of procaspase-3 includes an N-terminal prodomain and two subunits, one large and one small (referred to as p20 and p10, respectively). Together, these two subunits create the catalytically active pocket of the mature protease (Julien and Wells, 2017; Sakamaki and Satou, 2009).

In mammals, the 18 caspases identified have been subdivided based on their structure, function, and substrate specificity into two subfamilies commonly referred to as inflammatory and apoptotic caspases. Caspases -1, -4, -5, -11, -12, and -13 belong to the subfamily of inflammatory caspases that share a preference for the substrate sequence Trp-Glu-His-Asp (WEHD; Scott and Saleh, 2007). The apoptotic subfamily of caspases comprises two subgroups referred to as initiator/apical and effector/executioner caspases. Apoptotic stimuli trigger the activation of the initiator caspases (caspase-2, -8, -9, and -10), which then cleave and thereby activate the effector members (caspase-3, -6, and -7). The latter, in turn, target and cleave proteins that contain the Asp-Glu-Val-Asp (DEVD) sequence motif (Eckhart et al., 2008). Caspase-14 is distinct from both the inflammatory and apoptotic caspases by its lack of either of the features that define the inflammatory and apoptotic caspase subfamilies. Another more recently discovered four caspases, caspase-15, -16, -17, and -18, are not yet functionally well-characterized (Eckhart et al., 2008).

## Transcriptional regulation of caspase-3

The gene encoding human caspase-3 maps to q33-q35.1 on chromosome 4 and contains seven exons that comprise 2,635 base pairs. Two distinct forms of caspase-3 transcripts have been reported; the main transcript being 834 bp long, encoding a procaspase-3 protein composed of 277 amino acids (Végran et al., 2011). A shorter isoform referred to as caspase-3s that lacks amino acids encoded in exon 6 due to alternative splicing has also been identified (Huang et al., 2001) and can inhibit apoptosis, potentially through a direct interaction with procaspase-3 to block its proteolytic activation (Végran et al., 2011). Also of interest is the finding that the MCF7 human breast cancer cell line expresses only a truncated version of caspase-3 as a result of the 47-bp deletion in exon-3 that lacks the well-characterized proteolytic domain (Jänicke, 2009; Kagawa et al., 2001). Thus, other caspases (e.g., caspase 6 or 7) must be able to provide the general proteolytic function of caspase-3 required for its late role in apoptosis, and, any role that caspase-3 plays in MCF7 cells must be mediated features of its 5′ features. MCF7 cells thus serve as an important model for future interrogations of potential nonapoptotic roles of caspase-3.

The caspase-3 gene promoter contains several Sp1-like sequences, and its mRNA expression can be regulated by several transcription factors including Sp1 and p73 (Sudhakar et al., 2008). For example, cisplatin treatment of human cells results in an upregulation of caspase-3 transcript levels in a p73- and Sp1-dependent manner (Sudhakar et al., 2008). Reporter assay experiments also indicate Sp1 or Sp1-like proteins are required for the p73-induced activation of the caspase-3 promoter. Other transcription factors, including hypoxia-inducible factor 1α (HIF-1α; Van Hoecke et al., 2007), Stat3 (Silva et al., 2015), FOXO1 (Sudhakar et al., 2008), and c-Jun:ATF2 (Song et al., 2011) regulate the expression of murine caspase-3 by binding to its promoter. However, despite a significant similarity between the mouse and human caspase-3 promoters, direct evidence for the binding of the same transcription factors to the human caspase-3 promoter is lacking.

Caspase-3 appears to be ubiquitously expressed in normal tissues, but at variable levels. For example, an aging-associated epigenetic mechanism regulating caspase-3 expression was inferred from the finding that caspase-3 transcripts were reduced in older as compared with younger rat brain tissues and that this transcriptional silencing in older tissues was associated with an age-dependent increase in DNA methylation and decrease in histone 4 acetylation of the caspase-3 promoter (Yakovlev et al., 2010). In cancers, an increased expression of the procaspase-3 transcript and protein has been observed and linked to the dysregulation of the pRb/E2F pathway (Boudreau et al., 2019).

## Role of caspase-3 as an inducer of apoptosis

Cell death may be achieved by a variety of distinct mechanisms. During development and throughout life in cell renewal tissues, many cells are continuously eliminated by mechanisms that induce apoptosis (Fuchs and Steller, 2015; Negroni et al., 2015). The term “apoptosis” was coined by Kerr and colleagues in 1972 to distinguish between “naturally occurring” cell death that is part of normal development and necrosis caused by acute tissue injury (Kerr et al., 1972). An important role of apoptosis in animal tissues was later uncovered in pioneering studies of model organisms including *Caenorhabditis elegans* (Ellis and Horvitz, 1986), *Drosophila melanogaster* (Peterson et al., 2003; Wang et al., 1999), and inbred strains of mice (Fuchs and Steller, 2011; Nagasaka et al., 2010).

Apoptosis can be triggered both by interactions with extracellular factors and by intrinsic (intracellular) events. Extrinsic factors can be steroid hormones as well as various ligands of the tumor necrosis factor (TNF) receptor superfamily (e.g., FASL, TRAIL, TNF-α; Aram et al., 2017). Extracellular-mediated ligand binding to one of these death receptors causes caspase-8 to bind to the Fas-associated via death domain (FADD) adaptor protein, thereby forming a death-inducing signaling complex (DISC). Recruitment of caspase-8 to DISC facilitates its oligomerization and activation through self-cleavage. Cleaved caspase-8 then induces activation of downstream effector caspases, whose activities subsequently bring about the final stages of apoptotic cell death (Julien and Wells, 2017).

The intrinsic or mitochondrial pathway leading to apoptosis can be initiated by a variety of stimuli including viral infections, hypoxia, hyperthermia, oxidative stress, and intrinsically detected stress signals resulting from exposure to toxic chemical or radiation exposure, as occurs, for example, in cancer patients given chemotherapy or radiotherapy (Julien and Wells, 2017). A consequence of such pro-apoptotic cellular stressors is permeabilization of the mitochondrial outer membrane and release of apoptogenic factors such as cytochrome c from the mitochondrial intermembrane space into the cytosol. Subsequently, an apoptosomal complex (containing cytochrome c/Apaf-1/caspase-9) is formed which then triggers the activation of effector caspases, including caspase-3/7 (Julien and Wells, 2017). Similarly, reduced exposure of many cell types to certain external growth factors, cytokines, hormones, or cell–cell interactions on which their viability depends can also activate the intrinsic pathway of apoptosis, thus revealing a vital role that these external factors normally play in blocking the default activation of the apoptotic response machinery (Sakamaki and Satou, 2009). This latter role reflects the important evolutionary conservation of a mechanism to control normal cell numbers both during development and later under homeostatic conditions of tissue maintenance.

## Morphological and biochemical features of apoptosis

During apoptosis, activated caspase-3 cleaves a wide variety of downstream substrates that lead to typical morphological changes in apoptotic cells. For example, caspase-3-mediated cleavage of inhibitor of caspase-activated DNAse (ICAD) results in the activation of caspase-activated DNAse (CAD). This activation of CAD then induces chromatin condensation and DNA fragmentation by cleaving DNA at internucleosomal linker sites between the nucleosomes and the consequent generation of 180 bp fragments of DNA and multiples thereof (Enari et al., 1998; Larsen and Sørensen, 2017). Evidence of this form of DNA fragmentation is commonly detected by a DNA laddering assay (Smyth and Berman, 2002), a hallmark of cells undergoing apoptosis. A marker of an earlier stage of apoptosis is the externalization of phosphatidylserine from the inner layer of the plasma membrane. This allows phagocytes to recognize and phagocytose apoptotic cells and fragments in their vicinity (Shiratsuchi et al., 1998; Wu et al., 2006). Annexins are proteins that bind to the phosphatidylserine residues, and can thus serve as recognition ligands for such externalized forms of phosphatidylserine. Cell surface binding of Annexin V is thus now widely used to detect cells in the early stages of apoptosis. Other morphological changes in apoptotic cells include cell shrinkage and the formation of cytoplasmic blebs and the release of apoptotic bodies. Cleavage of other targets of caspase-3 (e.g., ROCK1) results in these and other dramatic morphological hallmarks, including cell shrinkage and actinomyosin contraction (Elmore, 2007).

## Non-apoptotic actions of caspase-3: Two mechanisms

Accruing evidence suggests that caspase-3 activities can also affect the survival, proliferation, and differentiation of both normal and malignant cells and tissues, in addition to its intrinsic function as a successful executioner of cell death. Additional ways in which caspase-3 (and other related caspases) is now known to affect the behavior of living cells and tissues as discussed below include both “nonautonomous” and “cell autonomous” (or direct) mechanisms of initiation and execution.

**Figure 1.**
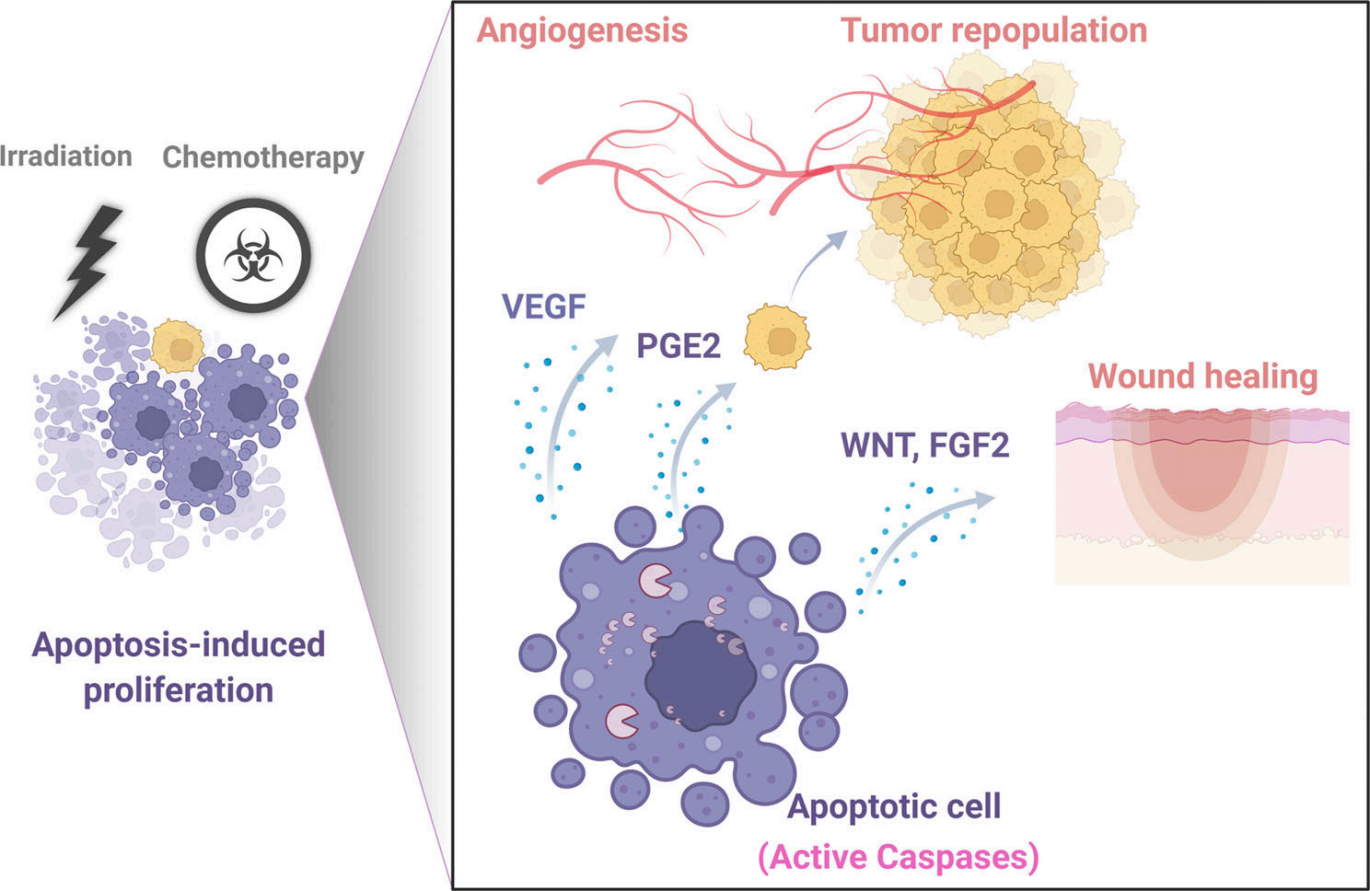
**Schematic illustrating caspase-dependent apoptosis-induced release of factors that then promote the proliferation of adjacent tumor and endothelial cells (Cheng et al., 2019; Feng et al., 2017; Kurtova et al., 2015; Lin et al., 2018; Tong et al., 2018).** Apoptotic cells promote the proliferation of adjacent cells through AiP. This process is mediated by active caspase-3, which activates different signaling pathways leading to enhanced angiogenesis, tumor repopulation, and wound healing.

**Figure 2.**
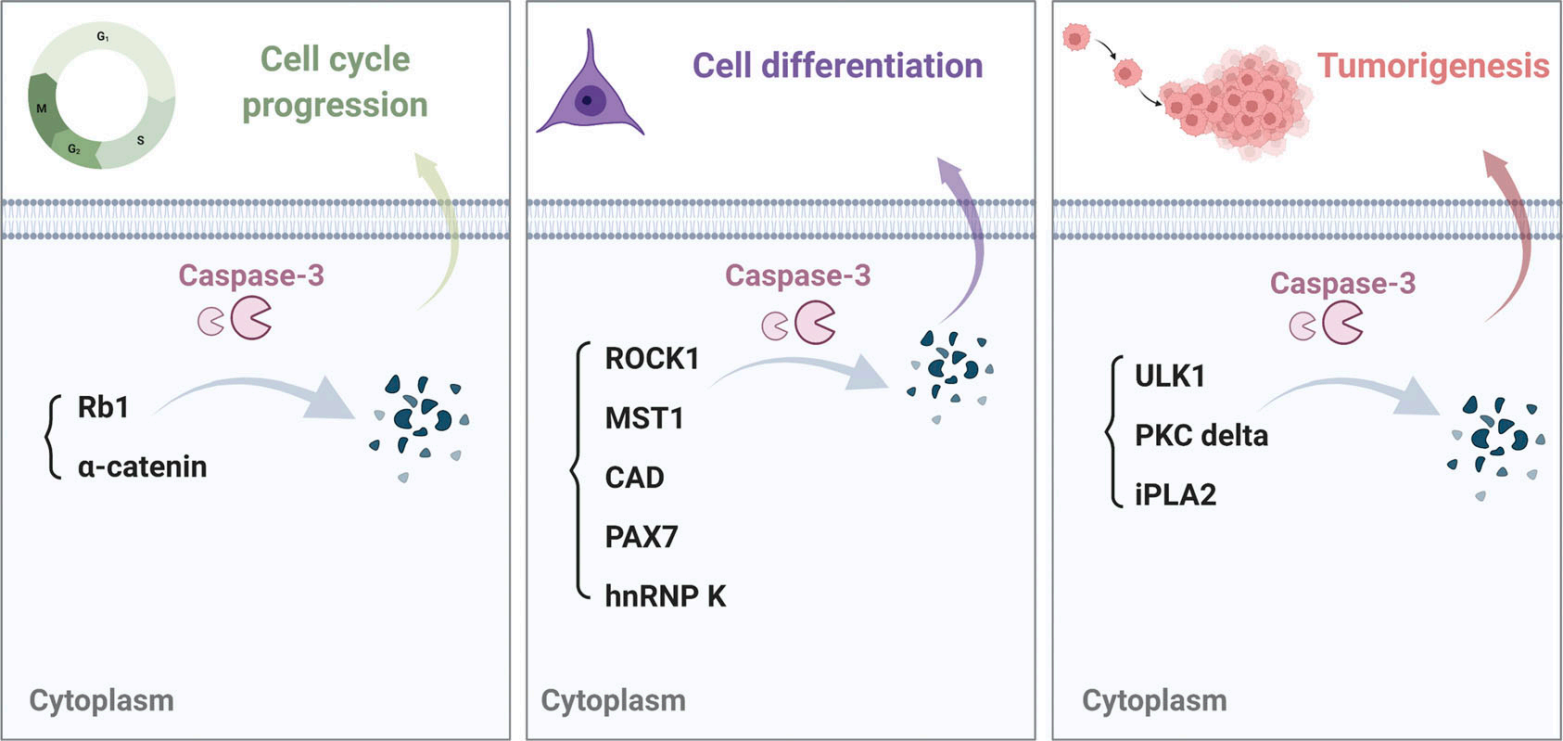
**Examples of mechanisms regulating cell cycle progression, differentiation, and tumorigenesis mediated by intrinsic caspase-3 cleavage of specific target proteins (Dick et al., 2015; Gabet et al., 2011; Larsen et al., 2010; Man et al., 2017; Naarmann-de Vries et al., 2013; Yosefzon et al., 2018).** Caspase-3 mediated proteolytic activity regulates a variety of cellular processes by the cleavage of downstream targets.

The term, nonautonomous as used here, refers to mechanisms that mediate the induction of compensatory proliferation of cells adjacent to other cells undergoing caspase-3-mediated apoptosis (Boland et al., 2013; Li et al., 2010b; Fig. 1). In contrast, the term, cell-autonomous (or direct) refers to intrinsically mediated activities of caspase-3 that do not result in cell death (i.e., that are incomplete and/or independent of paracrine effects on neighboring cells, see example in Fig. 2). In the latter case, caspase-3 is thought to interact directly with intracellular signaling pathways and ultimately gene expression profiles that elicit changes in stemness, differentiation, and the proliferative activity in the same cell in which the caspase-3 is expressed.

## Nonautonomous roles of caspases: Caspase-driven apoptosis-induced proliferation of adjacent cells

Multiple examples exist of activated caspase-induced apoptotic cells stimulating mitogenic signaling and proliferation of neighboring surviving cells. Such nonautonomous mechanisms of caspase-stimulated proliferation are referred to as compensatory proliferation or apoptosis-induced proliferation (AiP; Mollereau et al., 2013). AiP plays a critical evolutionary role in wound healing and tissue regeneration (Connolly et al., 2014; Fogarty and Bergmann, 2017; Ryoo and Bergmann, 2012). Most of our understanding of caspase-mediated nonautonomous proliferation and AiP signaling comes from genetic studies of *Drosophila* (Fan and Bergmann, 2008; Morata et al., 2011). Drosophila initiator (Dronc) and effector caspases (DrICE and Dcp-1) play critical roles in inducing compensatory proliferation through Jun N-terminal kinase (JNK) or Hedgehog signaling pathways in the eye and wing tissues (Fan and Bergmann, 2008). However, AiP also has an important role in tissue regeneration in other organisms. For example, in *Xenopus laevis*, a nonautonomous caspase-3 activity is required for tail regeneration (Tseng et al., 2007). Similarly, in planaria (flat worms), apoptosis is needed to support full tissue regeneration (Hwang et al., 2004). In hydra, AiP contributes to head regeneration following head amputation through Wnt3 signaling (Chera et al., 2009), and in mice, genetic models indicate that effector caspases in apoptotic cells can contribute to skin wound healing and liver regeneration through a so-named “Phoenix Rising” pathway (Li et al., 2010b). In mammals, caspase-3-mediated AiP contributes to a proliferation of hair follicle stem cells (HFSCs) and wound healing in caspase-9 deficient mice. Caspase-9-deficient HFSCs show an active apoptotic state with the accumulation of activated caspase-3. Mechanistically, this appears to be mediated by the caspase-3-promoted secretion of Wnt3 from adjacent apoptotic HFSCs due to the ability of caspase-3 to cleave dual specificity phosphatase 8 (Dusp8), which in turn, leads to the activation of p38-Mapk, and a consequent increase in the transcription and secretion of Wnt3 (Ankawa et al., 2021).

Taken together, these examples of AiP signaling suggest a conservation during the evolution of signaling mechanisms that enhance normal tissue regeneration. It is therefore interesting to speculate that the same or similar mechanisms might also contribute to the abnormal proliferation and deregulated growth of malignant cells. Indeed, evidence suggests that AiP is involved in tumorigenesis by contributing to tumor repopulation (Huang et al., 2011), therapeutic resistance (Kurtova et al., 2015; Lin et al., 2018; Tong et al., 2018), and angiogenesis (Cheng et al., 2019; Feng et al., 2017). In apoptotic cells, activated caspase-3 and -7 can cleave and activate the calcium-insensitive phospholipase A2 (iPLA2) leading to the production of prostaglandin E2 (PGE2), a potent inducer of AiP. Specifically, caspase-dependent PGE2 production induces the proliferation and regeneration of malignant cells following their exposure to radiation (Huang et al., 2011; Cheng et al., 2019; Donato et al., 2014) or chemotherapy (Bernard et al., 2019; Cui et al., 2017; Kurtova et al., 2015). Importantly, in addition to promoting tumor cell regrowth, caspase-3 stimulated PGE2 release contributes to epithelial–mesenchymal transition (EMT), anti-cancer immunity, and therapeutic resistance (Tong et al., 2018). Interestingly, several reports have also suggested the ability of inhibitors that target cyclooxygenase-2 (COX2)/PGE2 signaling to abrogate tumor repopulation or chemo-resistance. For example, in vivo administration of celecoxib (a COX2 inhibitor) attenuated chemoresistance in a xenograft tumor generated from a patient with bladder cancer (Kurtova et al., 2015). The administration of PGE2/EP4 antagonists in vivo also enhanced the chemosensitivity and blocked the growth of breast cancer (Lin et al., 2018) and melanoma (Zelenay et al., 2015). Although inhibitors of the COX/PGE2 axis have shown efficacy against tumor chemoresistance, AiP signaling does not rely exclusively on the COX/PGE2 pathway. Treated tumor cells might thus develop resistance by activating alternative mechanisms of AiP signaling. Accordingly, it could be important in the future to fully characterize the spectrum of signaling pathways that contribute to AiP in different cancer types.

Increased local production of mitogens followed by radiation or chemotherapy may not only enhance tumor regrowth and/or chemoresistance but may also promote angiogenesis and neovascularization (Feng et al., 2015). Notably, caspase-3-mediated signaling following radiation or chemotherapy has been found to increase VEGF production from treated cancer cells (Cheng et al., 2019; Feng et al., 2017) and thereby promote tumor regrowth by inducing angiogenesis (Bernard et al., 2019; Cheng et al., 2019; Feng et al., 2015, 2017). Interestingly, inhibition of caspase-3 activity showed efficacy against angiogenesis post-radiation or chemotherapy (Bernard et al., 2019; Feng et al., 2017). However, the general anti-tumor efficacy of inhibitors that target angiogenesis remains arguable as some tumor types appear capable of vasculogenic mimicry independent of angiogenesis (Linder and Tschernig, 2016; Wei et al., 2021). Nevertheless, it is interesting that the levels of cleaved caspase-3 in untreated melanoma tumors were found to correlate with evidence of such increased vasculogenic mimicry and metastasis (Vartanian et al., 2007). Moreover, the treatment of melanoma cells in vitro with a broad range caspase inhibitor (zVAD-fmk) and a more specific caspase-3 inhibitor (DEVD) blocked their migration, invasion, and capillary-like tube forming ability (Vartanian et al., 2007). Although AiP relies on caspase-3 activation in many of the above examples, AiP can also promote apoptotic resistance in surrounding cells through mechanisms that are independent of caspase-3 activation. For example, dying HeLa cells release FGF2, which can promote survival and apoptotic resistance in the surrounding cells. Importantly, in this case, the release of FGF2 by the Hela cells is dependent on their initiation of apoptosis, but not via caspase-3 activation. However, the treatment of the cellular targets of the released FGF2 with FGF-receptor inhibitors or the removal of their exposure to apoptotic inducers did sensitize cells to cytotoxic therapy and delayed wound healing (Bock et al., 2021).

## Cell-autonomous roles of caspase-3: Intrinsic regulation of cell proliferation

Sublethal activation of caspase-3 has been shown to regulate cell proliferation intrinsically by the proteolytic cleavage of a variety of substrates. Table 1 summarizes direct targets of caspase-3 and the outcome of their cleavage. Among caspase-3 targets are known regulators of cell cycle checkpoints (Li et al., 2010a). These substrates were first identified in apoptotic cells. However, further studies showed that the cleavage of these substrates by caspases regulates cell cycle progression even in the absence of apoptosis (Li et al., 2010a; Yosefzon et al., 2018). An interesting target tissue in this regard is the mouse epidermis (Yosefzon et al., 2018). In this case, the loss of caspase-3 decreases epidermal cell proliferation and reduces the size of the sebaceous gland. This implies a normal dependency of the maintenance of these cells on caspase-3. Caspase-3 also cleaves α-catenin in mouse epidermal cells and hence promotes the release of YAP from an α-catenin-YAP complex, thereby activating the ability of YAP to translocate into the nucleus and stimulate a proliferative response (Yosefzon et al., 2018). In proliferating HeLa cells, caspase activity was reported to act specifically at the M to G1 transition step (Hashimoto et al., 2011), and, in activated mouse T cells, changes in caspase-3 levels correlate with their timed entry into proliferation, as measured by the acquired expression of Ki67 (McComb et al., 2010; Misra et al., 2005). Conversely, treatment with caspase inhibitors suppresses proliferation (Misra et al., 2005). However, the detailed mechanisms by which activated caspase-3 acts to support cell cycle progression without causing cell death remain poorly understood.

**Table 1.** Summary of direct targets of CASPASE-3 in non-apoptotic settings

Protein	Cleavage outcome	Effect on cells	Cell type studied	Organism	Mechanism	Reference
PKC delta	Activation	Enhanced angiogenesis and tumor repopulation	Colorectal, pancreatic cancer	Human	Activation of AKT-VEGFA pathway	Cheng et al. (2019), Cheng et al. (2015)
ROCK1	Activation	Terminal maturation	Erythroblasts	Mouse	Phosphorylation of the light chain of myosin II	Gabet et al. (2011)
A-catenin	Inactivation	Increased cell proliferation and organ size	Epidermal cells	Mouse	Increased nuclear localization of YAP1	Yosefzon et al. (2018)
Retinoblastoma (Rb) protein	Inactivation	Nuclear reprogramming in iPSC induction	Fibroblasts	Human	Cell cycle progression by Rb inactivation	Li et al. (2010a)
iPLA2	Activation	Increased cell migration	Ovarian cancer cells	Human	Activation of AKT pathway	Zhao et al. (2006)
iPLA2	Activation	Increased tumor repopulation	Breast cancer cells	Mouse, human	Enhanced paracrine signaling	Huang et al. (2011)
MST1	Activation	Skeletal muscle differentiation	Myoblasts	Mouse	Activation of MAPK pathway	Fernando et al. (2002)
Caspase-activated DNase (CAD)	Activation	Skeletal muscle differentiation	Myoblasts	Mouse	Induction of DNA strand breaks	Larsen et al. (2010)
Pax7	Inactivation	Self-renewal of satellite cells	Satellite cells	Mouse	NA	Dick et al. (2015)
hnRNP K	Inactivation	Terminal maturation	Erythroblasts	Mouse	NA	Naarmann-de Vries et al. (2013)
ULK1	Inactivation	Leukemogenesis	Mouse fetal liver cells	Mouse	ULK1-dependant autophagy induction	Man et al. (2017)
DUSP8	Inactivation	Cell proliferation and wound healing	Hair follicle stem cells	Mouse	p38-Mapk -mediated Wnt3 signaling	Ankawa et al. (2021)

Effector caspases have also been found to play important roles in regulating the proliferation of mouse cardiac muscle cells. The hearts of caspase-3 and -7 double mutant neonatal mice are lighter than those of wild-type mice with cardiomyocyte hypertrophy and reduced numbers of myocytes (Cardona et al., 2015). Interestingly, the overexpression of inactive mutants of caspase-3 or -7 led to an upregulation of genes that were downregulated in caspase knockout myocardium, including genes regulating cell division. This novel finding strongly suggests that effector caspases might regulate cell proliferation in the mouse myocardium independent of their proteolytic activity (Cardona et al., 2015). Similarly, the proteolytic-independent activity of caspase-3 is important for the secretion of fibronectin and the in vitro adhesive and migratory properties of mouse embryonic fibroblasts (Brentnall et al., 2014). Taken together, these studies point to a novel role of effector caspases in regulating the proliferation and migration of mammalian cells that can be independent of their known proteolytic activities.

In contrast, mouse knock-out experiments show caspase-3 and -7 to be dispensable for normal development and subsequent maintenance of the intestine under steady-state conditions (Ghazavi et al., 2022). Interestingly, caspase-3^−/−^ mice display increased numbers of splenic B cells in vivo that also show a hyperproliferative response following in vitro stimulation (Woo et al., 2003). This has been explained by activated caspase-3-mediating the cleavage of p21 (the cyclin-dependent kinase [CDK] inhibitor) at its C terminus, thereby disrupting the ability of p21 to interact with proliferating cell nuclear antigen (PCNA) leading to cell cycle inhibition (Woo et al., 2003). A more recent example of the presence of active caspase-3 in the absence of apoptosis has been reported in our own studies of normal human mammary cells (Knapp et al., 2017). In these experiments, we found that cleaved caspase-3 could be detected in a purified fraction of freshly isolated viable human luminal progenitor cells. Moreover, those containing activated caspase-3 retained their ability to subsequently proliferate in response to epidermal growth factor (EGF) in vitro (Knapp et al., 2017). However, if or how this reflects a functional role of caspase-3 in regulating human mammary cells in vivo remains unknown. Taken together, these tissue-specific findings indicate that the determination of where and how caspase-3 plays a role in tissue modeling and maintenance in mammals is context-specific.

## Cell-autonomous roles of caspase-3: Regulation of cell differentiation

Activation of caspases can also have important effects on the differentiation and maturation of both human and mouse cell types, independent of any evidence of activation of apoptosis (Baena-Lopez et al., 2018; Fujita et al., 2008; Tang et al., 2012; Zermati et al., 2001). The majority of evidence for this has been obtained from comparative studies of wild-type and caspase-3 deficient mice as summarized in Table 2. For example, caspase activation regulates skeletal muscle differentiation by cleavage-mediated activation of Mammalian Sterile Twenty-like kinase (Fernando et al., 2002). Caspase-3-deficient mice also display abnormalities in osteogenic (Miura et al., 2004) and cardiac muscle differentiation (Cardona et al., 2015).

**Table 2.** Summary of the phenotypes of caspase-3 deficient mice

Genotype	Genetic background	Phenotype	Mechanism	Ref
Caspase-3^−/−^	B6.129S1	Reduction in total skeletal muscle mass; Myoblasts display a differentiation defect	Proteolytic function of caspase-3 activates MST1 and leads to myoblast differentiation	Fernando et al. (2002)
Caspase-3^−/−^	C57Bl/6	Deletion preserves hematopoietic stem cell pool but perturbs their differentiation without affecting cell viability	Caspase-3 alters signal transduction by limiting activation of the Ras-Raf-MEK-ERK pathway	Janzen et al. (2008)
Caspase-3^−/−^	C57Bl/6	Defects in skin wound healing and in liver regeneration	Caspase-3 stimulates production of PGE2, to promote cell proliferation and tissue regeneration,	Li et al. (2010b)
Caspase-3^−/−^	C57BL/6	Decreased incidence of chemically induced skin cancer	Caspase-3 activation of ENDOG enhances radiation-induced DNA damage and oncogenic transformation	Liu et al. (2015)
Caspase-3^−/−^	B6.129S1	Embryonic lethal, defective brain development	NA	Kuida et al. (1996)
Caspase-3^−/−^	C57BL/6	Increased renal lesions and mild splenomegaly	Increased expression of inflammatory Casp12, was observed in these Caspase-3 KO kidneys	Suzuki et al. (2020)
Caspase-3^−/−^7^−/−^ (conditional cardiac-specific KO mice)	C57BL/6	Hypoplastic neonatal heart with reduced number of cardiomyocytes	Caspase 3 and 7 regulate expression of genes involved in cell cycle independent of their proteolytic activity	Cardona et al. (2015)
Caspase-3^−/−^7^−/−^ (conditional intestinal-specific KO mice)	C57BL/6N	Normal intestinal development	NA	Ghazavi et al. (2022)
Caspase-3^−/−^	B6.129S1	Delayed ossification and decreased bone mineral density	Caspase-3 regulates the TGF-β/Smad2 signaling pathway and cellular senescence	Miura et al. (2004)
Caspase-3^−/−^	B6.129S1	Diminished cell proliferation and reduced sebaceous gland size	Caspase-3 cleaves α-catenin and facilitates activation and nuclear translocation of YAP1	Yosefzon et al. (2018)

Caspase-3 activation also plays a role in the derivation of embryonic and induced pluripotent stem cells (ESCs and iPSCs; Li et al., 2010a; Dejosez et al., 2008). Retinoblastoma (Rb) protein is one of the factors that act downstream of caspases, and its cleavage and inactivation by caspase-3 can facilitate nuclear reprogramming during the inductive phase of iPSC generation. However, these studies also suggest that caspase-3 regulates the loss of differentiation properties, primarily through its proteolytic activity by cleaving a spectrum of downstream targets.

Taken together, these findings underscore the diversity of nonapoptotic roles that effector caspases may play during normal tissue development and the promotion or reversal of differentiation programs.

## Cell-autonomous roles of caspase-3: Regulation of cell survival

High activation of the catalytic potential of caspase-3 is clearly a common initiator of cell death by apoptosis. However, low levels of caspase activity in mildly stressed cells can confer protection against cell death (Khalil et al., 2012). Examples include the finding that caspase-3-deficient or caspase inhibitor-treated mice display increased cell death and tissue damage when exposed to certain chemical or environmental stresses; e.g., those causing sunburns, cardiomyopathy, or colitis (Khalil et al., 2012; Vanli et al., 2014; Yang et al., 2004). Even in mildly stressed cells, low levels of caspase-3 activity can lead to the activation of the anti-apoptotic Akt kinase (through partial cleavage of RasGAP; Khalil et al., 2012), which then promotes cell survival by stimulating the mTOR (Porta et al., 2014) or NF-κB (Hussain et al., 2012) pathway. Activated Akt also prevents further amplification of caspase activity by inactivating proapoptotic molecules such as Bad (Uchiyama et al., 2004). These examples again support a nonapoptotic function of caspase-3 in cells that do not end up undergoing apoptosis following the initial activation of caspase-3 by pro-apoptotic stimuli.

## Cell-autonomous roles of caspase-3: Promotion of malignant properties

Internal oncogenic promoting events, as well as externally derived stimuli such as chemotherapy (Ichim et al., 2015) or irradiation (Liu et al., 2015) applied to established malignant cell populations can activate apoptosis in them. However, as noted above for effects on processes that regulate normal cell behavior, pro-oncogenic mechanisms can also be affected (Davis et al., 2003). These include internal responses to stress or cell damage, such as MYC activation (Cartwright et al., 2017), metabolic changes (Shirmanova et al., 2017), enhanced endonuclease-induced DNA double-strand breaks (DBSs) that enhance genome instability, and the activation of DNA nucleases such as CAD (caspase-activated DNase; Ichim et al., 2015) or ENDOG (endonuclease G; Cartwright et al., 2017; Liu et al., 2015). Specific examples of DNA damage and genomic instability potentiating the malignant transformation include the demonstrated enhanced E1A+KRAS-induced transformation of mouse embryonic fibroblasts treated with ABT-737 (a BH3-mimetic compound) or Q-VD-OPh-mediated suppression of BCL-xL expression (Ichim et al., 2015). Similarly, Liu et al. (2015) showed that caspase-3 expression is required for chemical-induced skin carcinogenesis and the radiation-induced oncogenic transformation of the human mammary immortalized but non-malignant MCF10A cells, and for AML1-ETO-induced leukemia in mouse hematopoietic cells (Man et al., 2017).

In the absence of external proapoptotic factors, cells with established malignant properties can acquire an increased level of spontaneous DNA double-strand breaks due to the activation of DNA nucleases (CAD and ENDOG) by sublethally activated levels of apoptotic caspases and their downstream targets (Liu et al., 2017). These then activate ataxia telangiectasia mutated (ATM) and the consequent selection of treatment-resistant cells (Ali et al., 2022), and/or the promotion of cells with more aggressive tumorigenic properties, such as enhanced motility and metastasis (Berthenet et al., 2020; Sun et al., 2017) via the activation of NF-κB and Stat3 (Liu et al., 2017). Sublethal mitochondrial activation of caspase-3 and CAD can also promote invasion and metastasis formation by increasing chromosomal misalignments and the generation of micronuclei (Haimovici et al., 2022).

These examples point to a significant role of caspases in promoting the initiation of transformation, aggressiveness of cancer cells, and adversely affecting conventional treatment responses of fully transformed cells.

## Evolutionary evidence of an early role of caspase-3 in protein quality control and autophagy

**Figure 3.**
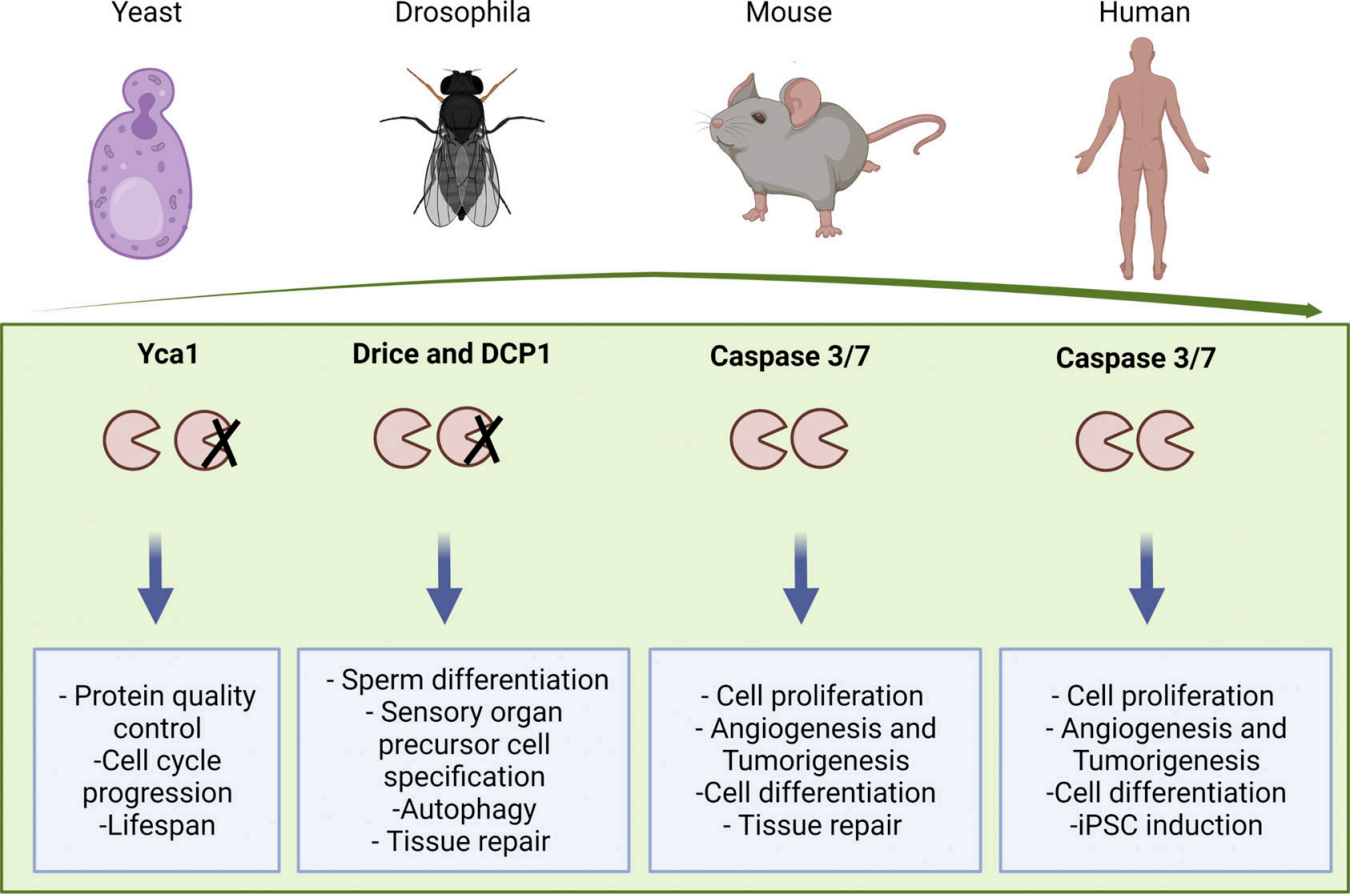
**Conservation of non-apoptotic cellular processes mediated by caspase from yeast to mammals.** Yeast Yca1 regulates protein quality control in catalytic and non-catalytic manners similar to catalytic and non-catalytic involvement of Drosophila effector caspases in autophagy.

The ability of apoptosis and its molecular mediators to control cell numbers rapidly and precisely during the development and homeostasis of tissues in multicellular organisms makes the survival advantages of acquiring and preserving a controlled apoptosis mechanism seem obvious from an evolutionary standpoint. However, caspase-like proteases are also found in unicellular eukaryotes. It thus seems likely that the first caspase-like genes to appear in evolution may have been selected originally for their ability to exert nonapoptotic functions that serve a primordial pro-survival role at the single-cell level and, only later acquired functions are important to more precisely control the development and survival of multicellular organisms (Fig. 3).

Like initiator caspases in mammalian cells, Yca1 (yeast caspase-1), also called MCA1 (metacaspase-1), from *Saccharomyces cerevisiae* regulates programmed cell death (PCD) in yeast. When yeast cells die as a result of stress or aging, they display apoptosis-like features such as nuclear fragmentation and loss of membrane integrity. However, despite these apparently functionally similar pro-apoptotic functions to mammalian caspases, yeast metacaspases have a different structure, mode of activation, and substrate specificity (Hill and Nyström, 2015). Indeed, the sequence homology of metacaspases and mammalian effector and initiator caspases (caspase-3 and caspase-9, respectively) is only 10%–11%. Moreover, metacaspases act as monomers and require calcium for their activation, unlike caspases which form dimers prior to their molecular activation. Their substrate specificities are also different; metacaspases cleave peptides after arginine and lysine residues, but caspases cleave after aspartate residues (Lee et al., 2008).

Yca1 is important for life-span extension in yeast, where it plays a role in the management and removal of protein aggregates that would otherwise be toxic to cell survival (Lee et al., 2010; Shrestha et al., 2019). Interestingly, Yca1-deficient yeast cells not only accumulate misfolded protein aggregates (Lee et al., 2010; Shrestha et al., 2019), they also exhibit a delay in cell cycle progression (Lee et al., 2008) and a shorter life span (Hill et al., 2014), both activities found to be operative in multicellular organisms, including mammals as noted above. Moreover, the role of Yca1 in protein quality control (PQC) is postulated to be mediated not only by the proteolytic degradation of protein aggregates, but also by acting as a protein scaffold for other PQC proteins or by managing protein misfolding through the ability of its Q/N-rich prodomain to participate in chaperone-like functions. These findings in yeast thus serve as important clues to understanding the nonapoptotic roles of caspases, including caspase-3, in more evolutionarily advanced organisms.

In eukaryotic cells, misfolded protein aggregates are cleared by two main mechanisms, both of which are controlled by caspases (DeVorkin et al., 2014; Hou et al., 2008; Lee et al., 2010; Shrestha et al., 2019). In addition to the ubiquitin-proteasome system (UPS; Lee et al., 2010), the autophagy–lysosome pathway degrades cellular contents and organelles to enable their recycling in response to cellular stress (Park et al., 2018). Autophagy could thus act as a pro-survival mechanism for cells under stress conditions by removing toxic metabolites, harmful protein aggregates, and damaged organelles leading to energy production for stressed cells. In *Drosophila*, its effector caspase (Dcp-1) regulates autophagy in the ovary where it activates autophagic flux in starved cells to protect them against the stress thus induced. Interestingly, this pro-survival role of Dcp-1 is mediated via a direct interaction with SesB, independent of the proteolytic functionality of Dcp-1 (DeVorkin et al., 2014; Hou et al., 2008). However, caspases can also regulate autophagy through their proteolytic activity, as many ATG proteins are known cleavage targets of caspases. For example, caspase-3-mediated cleavage of Beclin-1 and ATG4 inactivates autophagy and increases apoptosis in Hela cells and other cell lines (Betin and Lane, 2009; Zhu et al., 2010).

An additional example of a non-apoptotic role of procaspase-3 is its ability to regulate mitochondrial dynamics in degenerating dopaminergic neuronal mouse cell lines (Kim et al., 2018). In this case, the suppression of caspase-3 expression led to mitochondrial dysfunction, accumulation of damaged mitochondria, and downregulation of key transcriptional activators of mitochondrial biogenesis, such as Tfam and Nrf-1. Although the dependence of this non-apoptotic activity of caspase-3 on its catalytic potential was not specifically investigated, the treatment of these neuronal cells with a caspase-3 inhibitor did not affect Tfam or Nrf-1 expression, a finding consistent with the effects of caspase-3 suppression on the mitochondrial activity being independent of its proteolytic function. Of interest in this regard is the possibility that caspase-3 might be a therapeutic target for preserving degenerating neuronal cells in neurodegenerative diseases like Alzheimer's disease (AD), Parkinson's disease (PD), Huntington's disease (HD), and amyotrophic lateral sclerosis (ALS; Khan et al., 2015). However, these predictions are complicated by the evidence that caspase-3 can also contribute to the pathogenesis of AD and PD through its ability to cleave neuronal proteins such as amyloid precursor protein (APP) and Tau, an α-synuclein (α-Syn) and thereby influence the properties of the affected cells (Espinosa-Oliva et al., 2019). Caspase-3 can also cleave amyloid precursor protein (APP) to generate C31, a potent inducer of apoptosis in neural cells in vitro (Bertrand et al., 2001; Lu et al., 2000; Tsang et al., 2009), although results of corresponding in vivo studies are conflicting (D’Amelio et al., 2011; Harris et al., 2010). In addition, active caspase-3 may promote neural cell death via its ability to cleave Beclin-1 (Wang et al., 2017).

## Mechanisms that modulate caspase-3 activity

Importantly, for those situations where the proteolytic function of caspase-3 has positive effects on cell viability or other related activities, it seems likely that these are associated with a reduced induction of its proteolytic activity. Proteins known to have this capability include BID, NOXA, BIM, BAX, and BAK (Elmore, 2007; Fuchs and Steller, 2011). Similarly, mechanisms that upregulate anti-apoptotic protein levels, e.g., via Akt (Khalil et al., 2012) might be envisioned to serve as upstream regulators of this effect.

In situations where caspase-3 serves as a regulator of viable cell states, its catalytic activity at least needs to be maintained below the threshold level required for apoptosis. For example, during fly sperm individualization, expression of DrICE, the caspase-3 homolog, is maintained at a sufficiently low level to support caspase-3 degradation of the cytoplasmic contents needed for spermatid maturation, but insufficient to induce death by apoptosis. Two key proteins, Soti and dBruce, are important for the spatial regulation of caspase-3 activation that underpins the integrity of this process (Arama et al., 2003; Kaplan et al., 2010; Nakajima and Kuranaga, 2017). Interestingly, this spatial regulation of caspase activity seems to be a conserved mechanism, as the orthologous proteins of fly Soti and dBruce in mammals (i.e., ARTS) play key roles in the terminal differentiation of sperm in mice (Kissel et al., 2005). Another example of a non-lethal role of a caspase is seen during the process of sensory organ precursor cell specification in *Drosophila*. In this case, DmIKKε-mediated degradation of DIAP1, which in turn controls the temporal and transient activation of the *Drosophila* caspase DrICE, again enables caspase activities to be maintained at a sublethal level. still sufficient to support its non-apoptotic functions (Hashimoto et al., 2011). In mammalian cells, subcellular compartmentalization of caspases keeps their activation sufficiently reduced to support the progress of differentiation in erythroblasts (Zhao et al., 2016), keratinocytes (Weil et al., 1999), neural cells (Ertürk et al., 2014; Györffy et al., 2018), and embryonic stem cells (Fujita et al., 2008). Analogous mechanisms are also implicated in the process of IPSC formation from fibroblasts (Li et al., 2010a).

Caspase-3 activity can also be regulated by phosphorylation, ubiquitination, nitrosylation, and glutathionylation (Zamaraev et al., 2017). These posttranslational modifications (PTMs) cause conformational changes in caspase3 and can thus fine-tune its processing, enzymatic activity, and functions unrelated to its catalytic activity, including its ability to recruit other proteins. The phosphorylation site in caspase-3 that includes serine-150 (S150) is of particular interest because it is evolutionarily conserved, suggesting its likely importance in regulating apoptosis. For example, p38-MAPK-mediated phosphorylation of S150 in the active p20 subunits of caspase-3 in primary human neutrophils suppresses its enzymatic activity and reduces their apoptotic response (Alvarado-Kristensson et al., 2004). In contrast, in human monocytes protein kinase C-d (PKCd)-mediated caspase-3 phosphorylation induces a pro-apoptotic response by enhancing the activation of caspase-3 (Voss et al., 2005). In addition to protein kinases, protein phosphatases can regulate the activity of caspase-3. For example, the activation of protein phosphatase 2A (PP2A) during Fas-induced apoptosis results in dephosphorylation of both p38 MAPK and caspase 3, resulting in an increase in the catalytic activity of caspase-3 and the induction of apoptosis of neutrophils (Alvarado-Kristensson and Andersson, 2005). Phosphoproteomic analysis has further revealed that procaspase-3 phosphorylation at the +3 (P3) position of the DEVD sequence enhances its proteolytic cleavage by caspase-8 (Dix et al., 2012), thus highlighting the crosstalk that can affect downstream activities.

Caspase cleavage-site phosphorylation is another regulatory mechanism that inhibits caspase signaling by protecting substrates from caspase-mediated degradation (Tözsér et al., 2003). Casein kinase II (CK2), a ubiquitously expressed protein kinase in eukaryotes (Duncan et al., 2011), phosphorylates several pro-apoptotic proteins close to their caspase-3 cleavage site, thus protecting the cells from caspase-dependent degradation and apoptosis (Tözsér et al., 2003). The ability of CK2 to modulate caspase signaling and apoptosis is particularly important in the context of cancer biology as CK2 overexpression may promote cancer cell survival. At the same time, CK2 modulation of caspase-3 signaling also regulates vascular remodeling of the heart (Abdul-Ghani et al., 2017) and skeletal muscle (Dick et al., 2015). In the latter example, caspase-3 cleavage-mediated inactivation of Pax7 is a crucial step for terminating the muscle satellite cell self-renewal activity (Dick et al., 2015).

Ubiquitination of caspase-3 at its N-terminal domain is mediated by the Skp1–Cullin–F-box protein (SCF), an E3 ubiquitin ligase (Tan et al., 2006). Opposing this is the activity of ubiquitin-specific protease 15 (USP15), a deubiquitinating enzyme that counteracts the activity of the SCF complex and thereby increases the stability and activity of caspase-3 (Gewies and Grimm, 2003; Huynh et al., 2003; Xu et al., 2009). Inhibitors of apoptotic proteins (IAPs) that have a RING finger domain with an E3 ubiquitin ligase activity can also promote the degradation of caspases via the ubiquitin–proteasome pathway or by inhibiting the ability of caspase-3 to form functional dimers (Dumétier et al., 2020; Choi et al., 2009).

## Summary and future questions

In this review, we provide a summary of the extensive range of activities that caspase-3 has in viable cells that appear to be regulated and mediated by mechanisms distinct from those involved in the terminal execution of apoptosis. These findings raise interesting questions as to their context-specificity, origin, and relevance to the properties of malignant cells that remain unanswered. A growing body of data pertinent to this topic together with the knowledge that the forerunners of caspase genes and proteins originated as far back in evolution as yeast suggest the existence of as yet poorly defined mechanisms used by caspase-3 to exert important pro-survival roles in more complex, multicellular organisms including humans. The hallmark abilities of yeast precursors of caspases to regulate protein quality control and autophagy in more complex (insect) models may offer important clues to the future elucidation of the molecular pathways by which caspase-3 exerts, as yet uncharacterized, pro-survival roles in more advanced organisms, including humans and the malignant cells that arise within them.
